# Left-handed Surgical Instruments - A Guide for Cardiothoracic Surgeons

**DOI:** 10.1186/1749-8090-10-S1-A42

**Published:** 2015-12-16

**Authors:** Clare Burdett, Maureen Theakston, Joel Dunning, Andrew Goodwin, Simon Kendall

**Affiliations:** 1 Department of Cardiothoracic Surgery, James Cook University Hospital, Middlesbrough, UK

## Background/Introduction

For ease of use and to aid precision, left-handed instruments are invaluable to the left-handed surgeon. Although they exist, they are not available in many surgical centres. As a result, most operating theatre staff (including many left-handers) have little knowledge of their value or even application.

## Aims/Objectives

With specific reference to cardiac surgery, we explain how these instruments differ from right-handed ones, why they are essential and what is required in a 'standard set' - with tips on use.

## Method

This review draws on the knowledge and experience of left-handed Consultant surgeons, left-handed trainees and the theatre team.

## Results

Clear explanations with diagrams and photos are used to illustrate the differences of the most commonly used surgical instruments. We demonstrate how this affects needle and tissue handling, as well as performance. Tips are also given for scrub nurses on handling and loading left-handed instruments. We recommend a standard set that will allow Cardiothoracic Units to provide the essentials to any incoming left-hander.

## Discussion/Conclusion

For the left-handed surgeon, left-handed instruments are a pleasure to use. Subtle differences in design make a big difference to performance. This review is useful for left-handers, but also the right-handers that train and work with them.

